# Subgingival air polishing with trehalose powder during supportive periodontal therapy: use of a conical shaped tip during a randomized clinical trial

**DOI:** 10.1186/s12903-022-02109-1

**Published:** 2022-03-13

**Authors:** Anne Brigitte Kruse, Benjamin Jochen Wölki, Johan Peter Woelber, Eberhard Frisch, Kirstin Vach, Petra Ratka-Krüger

**Affiliations:** 1 Faculty of Medicine and Medical Center, Department of Operative Dentistry and Periodontology, University of Freiburg, Hugstetter Str. 55, 79106 Freiburg, Germany; 2 Northern Hessia Implant Center, Industriestraße 17A, 34369 Hofgeismar, Germany; 3 Faculty of Medicine and Medical Center, Institute of Medical Biometry and Statistics, University of Freiburg, Stefan-Meier-Strasse 26, 79104 Freiburg, Germany

**Keywords:** Air polishing, Subgingival biofilm removal, Subgingival instrumentation, Supportive periodontal therapy, Trehalose

## Abstract

**Background:**

This study investigated clinical parameters using a new air-polishing device compared to sonic scaling for subgingival biofilm removal during supportive periodontal therapy. The aim was to evaluate noninferiority of air-polishing compared to sonic scaling in deeper periodontal pockets with respect to pocket depth (PD).

**Methods:**

In 44 participants, 2 single-rooted teeth [(PD) ≥ 5 mm] were treated using a split-mouth design. While a new air polishing device with a conical shaped tip was used for the experimental group, sonic scaling was performed in the control group. PD, clinical attachment level (CAL), and bleeding on probing (BOP) were recorded at baseline, (T0) after 3 months (T1) and 6 months (T2). Pain perception was rated using a visual analog scale (VAS; 0 = no pain, 100 = maximum pain).

**Results:**

PD and CAL decreased significantly for both groups, while no intergroup differences were found (PD [mean, mm] control T0 5.96, T2 4.75; experimental T0 5.96, T2 4.8; intergroup *p* = 0.998; CAL [mean, mm] control T0 7.38, T2 5.84; experimental T0 7.28, T2 6.34; intergroup *p* = 0.368). For BOP, no intergroup differences were found from T0 to T2 (reduction control 42.5%; experimental 46.5% *p* = 0.398). Pain perception was significantly lower for air polishing (VAS [mean, mm] control 28.8, experimental 12.56; *p* = 0.006).

**Conclusion:**

None of the two treatment procedures showed inferior clinical effects with regard to PD, CAL and BOP with air polishing being more comfortable to patients.

*Trial registration* The study was registered in an international trial register on August 14/08/2019, before the start of recruitment (German Clinical Trial Register number DRKS00017844).

## Background

Air polishing has become an established procedure for the removal of subgingival biofilm during supportive periodontal therapy (SPT). Comparable clinical and microbiological results have been demonstrated in several studies with regard to standard procedures such as ultra/sonic instrumentation or the use of hand instruments [1–3]. A recent systematic review including 13 clinical studies confirmed a comparable efficacy in the control of biofilm and reduction of periodontal inflammation [4]. The main advantages of subgingival air polishing are lower abrasiveness and less time required for treatment [5]. In addition, the method was rated as more comfortable compared to alternative methods (hand instruments or sonic scaling) by the majority of patients in several studies [2, 3, 6, 7].

The use of air polishing for subgingival instrumentation during a clinical trial was first described by Petersilka et al. [8, 9] during two clinical trials. Here, a conventional air polishing device was used with glycine powder, and the beam was pointed into periodontal pockets in the apical direction. It was shown that in pockets of 3–5 mm, air polishing was superior to hand instrumentation with respect to the reduction of subgingival biofilm looking at CFUs from plaque samples. A study by Flemmig et al. [10] also used glycine powder and pointed the beam into the periodontal pocket [1]. Here, the efficacy of the treatment was evaluated by extracting and staining the study teeth immediately after instrumentation. As a result, sufficient removal of subgingival biofilm could be observed for smaller pockets, ≤ 3 mm. Subsequently, Moëne et al. [7] introduced a new air-polishing device using a triangular-shaped nozzle with different sideways orientations for water and the air-powder beam to reach deeper periodontal pockets and to reduce the risk of emphysema. Using this special device in combination with a low-abrasive glycine powder, a reduction of bleeding on probing (BOP) and subgingival plaque (CFUs) could be shown for pockets up to 9 mm over a period of 7 days after subgingival instrumentation. During another clinical trial published in 2014, which was assessed in patients during SPT with PD of 5–9 mm, the use of the aforementioned device combined with erythritol powder was able to show comparable reduction of periodontal pockets and bleeding on probing compared to the use of ultrasonic scaling over a period of 12 months [3]. In addition to erythritol and glycine, the disaccharide trehalose is one of the known low-abrasive powder substances, for which comparable efficacy has been shown in clinical studies [2, 4, 11].

Since evidence for low-abrasive powders has grown over the last years, data from clinical studies on different devices are scarce. In contrast to the increase in the range of products on the market, there are hardly any clinical studies on the use of new types of devices. In addition to the properties of the powder used and treatment factors such as duration, distance to the tooth surface and angle, the configuration of the air polishing unit plays a key role in the degree of abrasiveness and effectiveness of biofilm removal [5]. This topic is important not least because the subgingival use of compressed air is always associated with the risk of provoking emphysema, especially in areas of inflammation or a narrow zone of attached gingiva [12]. The development of new nozzles and tips for subgingival use is aimed at achieving optimum redirection of the compressed air to the root surface, even in deep periodontal pockets up to 9 mm. This reduces the risk of emphysema, contrary to an apical streaming direction, and thus promotes the safety of the application. At the same time, it might lead to increased effectiveness by pointing more directly on the root surface. Accordingly, more trials are needed investigating different devices for subgingival air-polishing and comparing different procedures.

The aim of this clinical study was to evaluate the use of a newly designed air polishing handpiece with a conical-shaped tip and a round cross section in the use of subgingival instrumentation compared to sonic scaling during SPT. Furthermore, the noninferiority of this treatment for PD was evaluated. Additionally, the pain perception for each method was assessed.

## Materials and methods

### Ethics approval and informed consent statement

This clinical trial was conducted in accordance with good clinical practice guidelines and respected the principles of the Declaration of Helsinki on human experimentation. The Ethical Committee of the University Medical Center Freiburg approved the study protocol with a positive vote (EK No. 188/19). All enrolled participants gave their written informed consent on study participation and signed a data privacy statement. This report follows the criteria of the CONSORT statement [13].

The study was registered in an international trial register on 14/08/2019, before the start of recruitment (German Clinical Trial Register number DRKS00017844).

### Study design

The study was conducted as an examiner-blinded randomized clinical trial over 6 months using a split-mouth design. Two nonadjacent single-rooted teeth with a periodontal pocket (5–9 mm) from the same jaw were chosen to serve either as the test or control group. While the test group received subgingival instrumentation using a new air polishing device with a conical shaped tip, the control group was treated using sonic scaling. While one blinded clinician (ABK) performed all measures, a second clinician (BJW) did subgingival instrumentation using different procedures.

It was hypothesized that treatment with the newly developed air polishing device and application tip shows noninferiority for the parameter pocket depth (PD) compared to treatment with a sonic scaler during SPT. The primary endpoint was the change in PD after 6 months. Secondary endpoints were the change in clinical attachment level (CAL), reduction in BOP, the need for retreatment, and pain perception for each method using a visual analog scale.

### Recruitment

Fifty participants were recruited from patients within regular supportive periodontal treatment at the Department of Operative Dentistry and Periodontology, Faculty of Medicine and Medical Center, University of Freiburg, Germany. Recruitment took place between August 2019 and July 2020.

### Inclusion and exclusion criteria

The inclusion of participants was based on the following criteria: two nonadjacent teeth in one jaw but different quadrants with persisting periodontal pockets (PD ≥ 5 mm and BOP + or ≥ 6 mm, and < 10 mm), regular SPT (attending their regular SPT sessions depending on their individual periodontal risk), and periodontitis grade A or B. A maximum of 30% smokers were included. The exclusion criteria were systemic antibiotic treatment within the study period, periodontal abscesses, suppuration from periodontal pockets, extraction of included teeth, severe systemic diseases such as HIV, cancer or poorly controlled diabetes mellitus, hemophilia or anticoagulation therapy, bisphosphonate antiresorptive therapy, and pregnancy.

### Clinical examination

A full-mouth plaque control record (PCR) [14] and modified sulcus bleeding index(SBI) [15, 16] were collected. For both designated teeth, PDs, CAL and BOP at 6 sites were recorded at baseline to determine the site with the deepest PD in mm and a pressure of approx. 0.2 N using a PCP UNC 15 periodontal probe (Hu-Friedy, Chicago, Illinois, USA). At these sites, follow-up examinations were recorded at 3 and 6 months for PD, CAL, and BOP. CAL was measured by recording PD and adding the distance from the gingival margin to the cementoenamel junction in the presence of recessions or subtracting it in the presence of swellings or hyperplastic gingiva.

### Intervention

In each study participant, one tooth was treated with air polishing (experimental group) and one tooth with sonic scaling (control group) at baseline. Clinical follow-up examinations were performed after 3 and 6 months. If the respective teeth showed PD > 4 mm or 4 mm and BOP + [17], treatment was repeated after 3 months using the respective treatment (air polishing or sonic scaling) and after 6 months using sonic scaling (Fig. 2). The need of retreatment was recorded at each stage and is shown in Table 2. Patients did not receive further oral hygiene instructions during the study period as they were already very well trained in the use of oral hygiene products and systematic cleaning within the current phase of SPT.

#### Experimental group

**Fig. 1:**
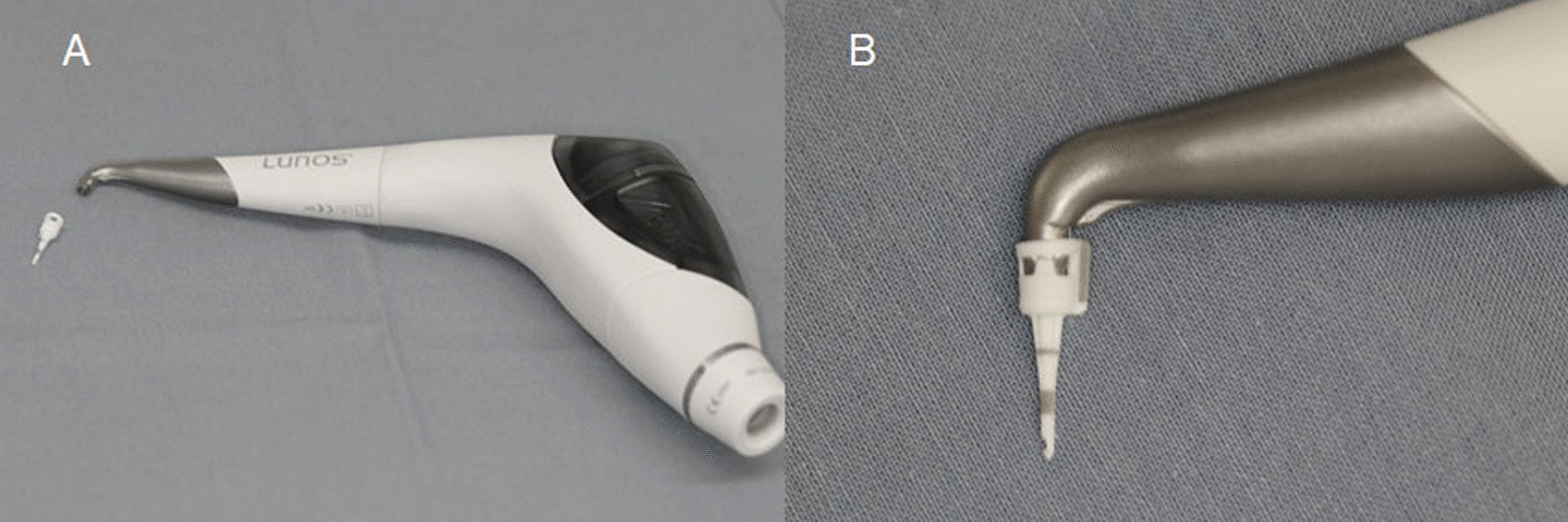
**a** Air-polishing handpiece with perio nozzle and powder chamber. **b** Conical shaped tip with markings at 3.5, 5.5 and 8.5 mm

The experimental group was treated with a newly designed air-polishing application tip (Perio Tip^®^, Dürr Dental SE, Bietigheim-Bissingen, Germany; Fig. 1a) in combination with a MyLunos^®^ air-polishing handpiece with Perio nozzle (MyLunos^®^Perio air-polishing handpiece, Dürr Dental SE, Bietigheim-Bissingen, Germany; Fig. 1b) and trehalose powder (MyLunos^®^ Perio Combi, Dürr Dental SE, Bietigheim-Bissingen, Germany) for 5 s at the assigned site. The conical shaped tip with a round cross section shows black markings at 3.5, 5.5 and 8.5 mm. While the air-powder beam leaves the tip through a single lateral opening on the distal aspect, water runs out of 6 perforations above the highest mark of 8.5 mm. The air-powder spray corridor was limited to the distal and apical directions at a 45-degree angle. The tip is additionally protected from being pulled off by a safety ring, which also ensures a single use only, since it breaks when the tip is taken off the device. The aforementioned handpiece contains a powder chamber with a filling capacity of ~ 18 g trehalose powder (Fig. 1b).

#### Control group

The control group received subgingival instrumentation using a sonic device, for at least 5 s at the assigned site depending on the extent of the root surface to be treated (SONICflex™ with tip Perio long, KaVo, Biberach/Ris, Germany).

#### Pain perception

Shortly before the upcoming treatment at baseline, the participants were informed that they would be asked to give an assessment of the painfulness immediately after the treatment. Their discomfort for each procedure was assessed by using a visual analog scale (VAS) from 1 to 10 (Price et al. [18]). The assessment of pain perception was not repeated at other time points, even if retreatment was necessary.

### Randomization, blinding and statistical analysis

All baseline and follow-up examinations were performed by the same experienced calibrated dentist blinded to the therapy (ABK). In the course of an intra-examiner calibration process prior to the study, a reproducibility of 100% was achieved tolerating a standard deviation of ± 1 mm. Treatment assignment for the two test teeth was randomized by a statistician (KV) by computer generating a randomization list. Based on this list, subgingival instrumentation was performed exclusively by a second dentist (BJW), who found the assigned treatment for each tooth in sealed envelopes from the statistician. The order of the teeth to be treated was based on the quadrant regardless of the procedure they were assigned to. Thus, teeth from the 1st and 3rd quadrant were treated before teeth from the 2nd and 4th quadrant.

The study was planned as a noninferiority trial for PD based on the results of a former study [2]. For the main outcome parameter PD, a noninferiority threshold of 0.4 mm was considered to be appropriate; a previous study showed that a standard deviation of 0.8 is realistic. This results in a sample size of 50 patients with a total of 100 teeth (50 teeth per group) with a power of 80% and using a 90% confidence interval. The test for noninferiority was performed for data after 6 months. Here, the air polishing device is considered noninferior if the lower value of the 90% confidence interval determined by means of the mixed linear model is above the lower noninferiority confidence limit. For descriptive analyses, relative frequencies, medians, means and standard deviations were computed. For within-group comparisons depending on the distribution of the data, paired t-tests, Wilcoxon matched-pairs signed-rank tests and McNemars tests were used. Linear mixed models with adjustment for sex, age, smoking status and the corresponding baseline value were used to evaluate device differences for changes from baseline to the 6-month investigation. Corresponding logistic mixed models were used for binary data. Statistical analysis was performed per protocol using STATA software (StataCorp LT, College Station, TX, USA, Version 16.1).

## Results

**Fig. 2:**
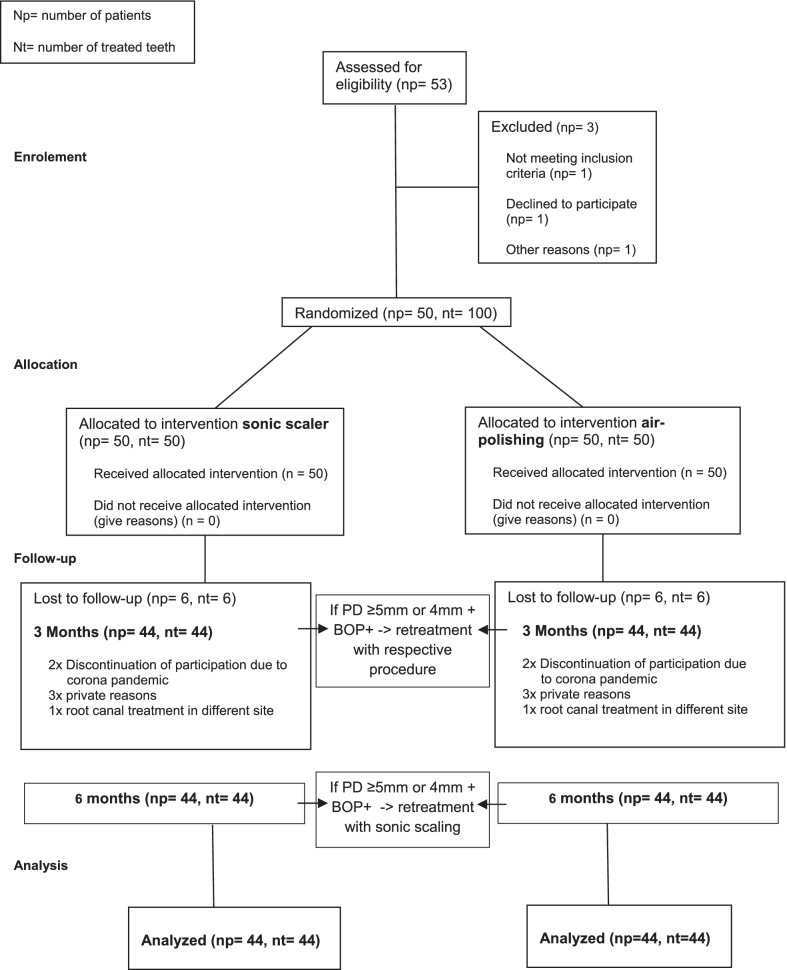
CONSORT flow diagram

Fifty participants met the inclusion criteria, gave their informed consent to participate in the study and received examination and treatment at baseline. After 6 months, clinical data from 44 participants were collected for analysis, while 6 were lost to follow-up for different reasons (Fig. 2). Due to coronavirus pandemic restrictions, for one participant, no 3-month data were collected, and only the 6-month follow-up could be carried out. For the same reason, another patient only received a 3-month follow-up. This results in a case number of 44 at both follow-up times. Furthermore, sensitivity analyses were carried out and led to almost identical results, especially without changes for statistical significance. For demographic data at baseline see Table 1.

**Table 1:** Demographic data

	n	%
*Gender*		
Male	30	60
Female	20	40
*Ethnic group*		
Caucasian	50	100
Other	0	0
*Smoking status*		
Non-smokers	36	72
Smokers	14	28

	n	P50	Mean	SD	Min	Max
Age (years)	50	61.5	61.72	10.99	35	87

*P50* median, *sd* standard deviation, *min* minimum, *max* maximum

### Pocket depths and clinical attachment level

**Fig. 3:**
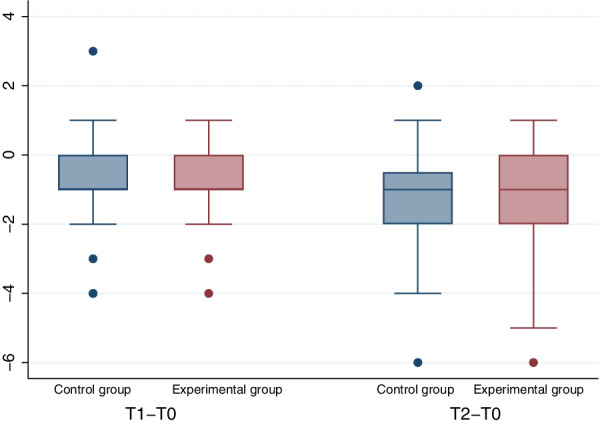
Boxplots for differences in PD (mm)

**Fig. 4:**
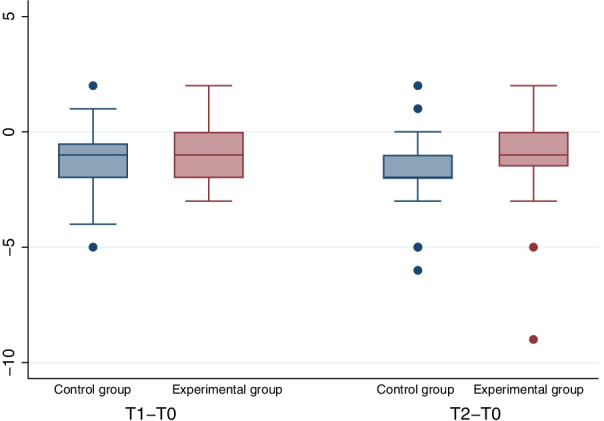
Boxplots for differences in CAL (mm)

Mean PD and CAL decreased for both groups significantly with a *p* ≤ 0.005 for all comparisons (PD T0 vs. T1, control − 0.86 mm, experimental − 0.86 mm; T0 vs. T2, control − 1.23 mm, *p* T0 vs. T2, experimental − 1.16 mm, *p*; CAL T0 vs. T1, control − 1.02 mm, experimental − 0.80 mm, T0 vs. T2, control − 1.50, p, experimental − 0.86 mm) without any significant intergroup differences (PD T0 vs. T2 *p* = 0.983; CAL T0 vs. T2 *p* = 0.358; Table 2 and Figs. 3, 4). There were no significant group differences for PD or CAL in the analyses of changes from T0 to T2 when adjusting for smoking status, sex, age and corresponding value at baseline. After 6 months (T2), 8 healthy periodontal sites (PD ≤ 3 mm) were found in the control group (n = 44), and 9 healthy periodontal sites were found in the experimental group (n = 44). For PD, a group difference of 0.0007 (90% CI [− 0.360, 0.362]) was found; hence, noninferiority of the air-polishing treatment was shown.

**Table 2:** Main clinical results

	Control group						Experimental group					
	Time	N	Median	Mean	Sd	∆ (sd)	Time	N	Median	Mean	Sd	∆ (sd)
PD (mm)	T0	50	6	5.69	1.05		T0	50	5.5	5.96	1.12	
	T1	44	5	5.11*	1.73	− 0.86 (1.25)	T1	44	5	5*	1.46	− 0.86 (0.98)
	T2	44	4	4.75**	1.67	− 1.23 (1.34)	T2	44	5	4.8**	1.5	− 1.16 (1.34)
CAL (mm)	T0	50	7	7.38	1.78		T0	50	7	7.28	1.99	
	T1	44	6	6.25*	2.09	− 1.02 (1.27)	T1	44	6	6.32*	1.95	− 0.79 (1.09)
	T2	44	6	5.84**	2.20	− 1.50 (1.47)	T2	44	6	6.34**	2.19	− 0.86 (1.80)
VAS (mm)	T0	50	21	28.75^§^	23.58		T0	50	8	12.56^§^	14.43	

BOP			% Positive			% Positive
	T0	50	90.0	T0	50	98.0
	T1	44	52.3*	T1	44	59.1*
	T2	44	52.3**	T2	44	52.3**

Need for retreatment			% Positive			% Positive
	T1	44	90.9	T1	44	86.4
	T2	44	75.0	T2	44	81.8

*PD* pocket depth, *CAL* clinical attachment level, *VAS* visual analog scale [0–100 mm, 0 no pain/100 maximum pain]; T0 = baseline, T1 = 3 months, T2 = 6 months; sd = standard deviation; ∆ = difference to T0; ^§^ intergroup difference *p* < 0.05 (Wilcoxon matched-pairs signed-rank test, McNemar for dichotomous variables), intragroup difference *p* < 0.05 (Wilcoxon matched-pairs signed-rank test, McNemar for dichotomous variables) T0 versus T1(*) and T0 versus T2(**)

### Bleeding on probing

For both groups, a reduction of BOP was found comparing T0 and T1 as well as T0 and T2 (proportion who were BOP positive at T0 and negative at T2 control 42.5%; experimental 46.5%, *p* < 0.0001 both groups). There were no group differences from T0 to T2 (intergroup *p* = 0.398; Table 2). The logistic mixed model computed an odds ratio for a site to be BOP positive after 6 months of 1.437 (95% CI 0.62; 3.333) for the experimental group in comparison to the control group, with no significant differences between the groups (*p* = 0.398).

### Need for retreatment

Both groups showed a decrease in the percentage of teeth that needed retreatment over the study period (Table 2). The need for retreatment after 3 months (T1) and 6 months (T2) did not differ significantly between groups (T1 *p* = 0.688; T2 *p* = 0.581). Additionally, for both groups, there was no significant difference in the number of teeth that needed retreatment once (*p* = 1.000) or twice (*p* = 0.791) during the study period (Table 2).

### VAS

**Fig. 5:**
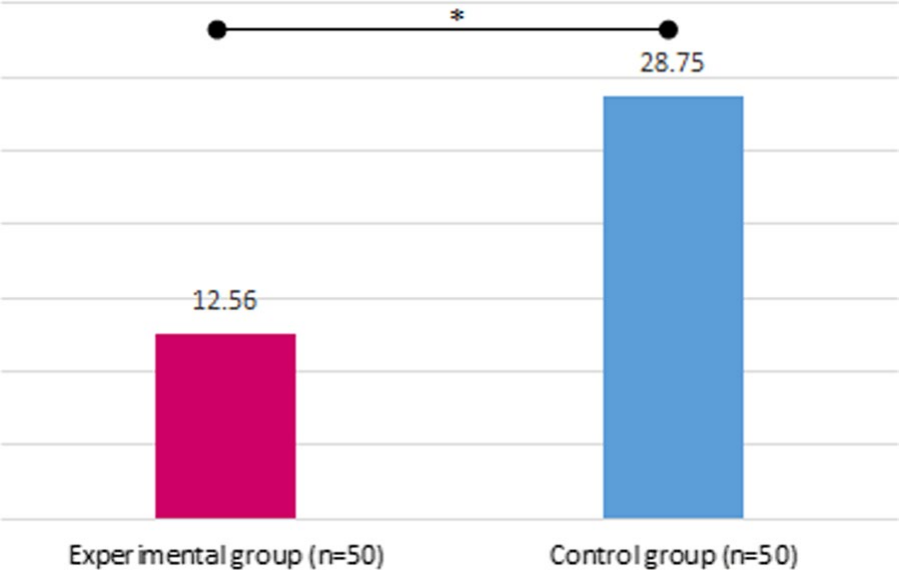
Pain perception using a visual analog scale (1–100 mm); values as means, *p < 0.005 (based on Wilcoxon matched-pairs signed-rank test)

The assessment of patients’ pain perception using a visual analog scale from 1 to 100 mm (0 = no pain; 100 = maximum pain) showed a significantly lower pain perception for the experimental group using air polishing compared to the control group using a sonic scaler (*p* = 0.006, Table 2 and Fig. 5).

### Plaque control record and sulcus bleeding index

The plaque control record (PCR) and sulcus bleeding index (SBI) were measured as a full-mouth status evaluation and did not change significantly (*p* = 0.067) from baseline (T0) to 6 months (T2). PCR decreased from a mean of 32.1% (T0, ± 20.2) to 26.7% (T2, ± 22.3), *p* = 0.067. SBI decreased from a mean of 33.2% (T0, ± 19.8) to 28.4% (T2, ± 21.0), *p* = 0.086.

No adverse effects caused by the use of either procedure (e.g., subcutaneous emphysema, periodontal abscess) were observed during the study period.

## Discussion

The use of the new air polishing device and a conical shaped application tip for subgingival instrumentation during supportive periodontal treatment was found to show no significant differences between groups in the reduction of PD as the primary outcome. Furthermore, the noninferiority of the application was confirmed for this parameter. Both treatment modalities showed a reduction with regard to PD, CAL, BOP, and the need for retreatment over 6 months. These findings correspond to several clinical trials in the field of subgingival air polishing during maintenance therapy [2, 3, 19, 20]. A recent systematic review summarized and confirmed the efficacy of air polishing treatment in controlling biofilm and periodontal inflammation after evaluating 13 randomized clinical trials [4]. Within this study, significant improvements in PD were observed in both groups. Compared to other studies using air polishing, here the reduction in PD with a mean of 1 mm after 6 months appears to be rather high [4]. However, comparable reductions can also be found in the literature [20, 21]. This could be explained by the fact that the two highest probing depths were selected and treated, re-examined after 3 months, and treated again if the probing depth was still high. It could be shown that a closer treatment interval might help to reduce PDs and promotes periodontal stability [22]. Moreover, deep PDs generally show greater reductions in PD compared to low PDs [23]. Thus, the close follow-up and retreatment as well as the presence of a very high probing depth at baseline could explain the supposedly high reduction.

Furthermore, regarding the measurement of pain perception, participants significantly felt more comfortable when air polishing was used. These findings confirm the broad consensus in the literature that air polishing is more comfortable for patients than alternative procedures such as hand instrumentation or ultrasonic instrumentation [4, 6]. However, if this effects clinical impact remains questionable.

This study adds new evidence to the field of air polishing devices that are designed especially for use in deeper periodontal pockets (5–9 mm) where surgical and regenerative therapeutic approaches would also be appropriate [24, 25]. To the authors' knowledge, all comparable clinical trials have been conducted with the same product thus far (Air Flow^®^ Master using the Perio-Flow^®^ Nozzle, EMS Electro Medical System S.A., Nyon, Switzerland) [4]. Although there are several new developments found on the market regarding subgingival instrument tips, there is no evidence for their use in clinical trials. One advantage of the new tip used in this study is its round and small diameter, which allows the tip to easily enter the periodontal pocket. However, the fact that there is only one opening for the powder-air beam at the distal aspect of the tip proved to be a disadvantage in handling. Posterior areas in particular are difficult to reach at distal sites and require a strong rotation of the user’s wrist.

The strength of this study is the use of a split-mouth design, which leads to a low risk of bias in patient-related factors such as smoking or other factors modulating the immune response when comparing the test and control groups. However, the fact that only one tooth with the highest probing depth per side was selected and followed up is a limitation of the study. Due to the particular clinical relevance of deep probing depths within SPT, it seemed reasonable to focus on these sites. Furthermore, a possible carry-over effect due to repeated treatment of the same sites cannot be excluded.

The fact that the study plan originally aimed at a maximum of 20% smokers but ultimately included 30% may appear to be a source of bias. However, the smoking population in Europe is reported to be 28% on average [26]. Thus, the proportion of smokers among the study participants appears to be representative.

Furthermore, it might be of concern that the study participants did not receive additional oral hygiene instructions during the study period. However, clinical parameters like PCR and SBI showed ad decrease over the study period.

Another clear limitation of this study is the short duration of the investigation. As seen in the systematic review mentioned before, in 9 out of 13 included studies, follow-up ended in less than 180 days. To date, only one retrospective analysis included follow-up examinations over 5 years during SPT [27]. Here, moderate periodontal pockets of mainly < 6.5 mm were treated using glycine powder and a conventional air polishing device without a special tip for subgingival use. Compared to only mechanical instrumentation using sonic scaling and curets there were found equivalent results for the reduction of PD. On the other hand, since the most comparable studies which included deeper periodontal pockets during SPT showed similar follow-up examinations at 3 and 6 months, the results of this study consolidate the evidence that could be found thus far. However, more long-term studies are needed to evaluate the effect over a longer period of time in deeper periodontal pockets. Furthermore, the addition of the assessment of oxidative stress biomarkers would be interesting in future studies [28, 29].

SPT is an important prerequisite for the success of periodontal therapy, as shown in several fundamental studies and a current systematic review [30–34]. In the context of evidence-based medicine, it should be noted that the recommendation of regular SPT has been included in the S3 level of the clinical practice guidelines previously released by the European Federation of Periodontology [25]. When performing regular subgingival instrumentation over a long-term period, it is important to use procedures that ensure effective biofilm control and simultaneous low substance loss of the dental hard tissue. In-vitro studies comparing the loss of root substance during different subgingival instrumentation procedures indicate that there is greater damage to the hard tissue when hand instruments or sonic scaling are used [35–37]. Complementary to this, comparable results are found for soft tissue damage according to different procedures [38]. Therefore, air polishing as a treatment method appears to fulfil the current requirements for effective and gentle subgingival biofilm control within SPTs. For regular and repeated use over years, however, long-term studies are pending and should also be carried out with respect to hard substance loss.

## Conclusion

For the primary outcome parameter PD noninferiority was shown for both treatment modalities. Secondary outcome parameters (CAL, BOP, need for retreatment) did not show significant differences for the use of a new air polishing device with a conical shaped tip for subgingival periodontal treatment compared to sonic scaling during SPT over a period of 6 months. Pain perception was significantly lower when air polishing was used.

## Data Availability

The datasets are available from the corresponding author on reasonable request.

## Abbreviations

BOP: Bleeding on probing
CAL: Clinical attachment level
CFU: Colony forming units
PCR: Plaque control record
PD: Probing depth
SBI: Sulcus bleeding index
SPT: Supportive periodontal treatment
VAS: Visual analogue scale
